# Traditional Chinese medicine for lupus nephritis: modulation of autoimmune pathogenesis

**DOI:** 10.3389/fphar.2025.1523272

**Published:** 2025-05-02

**Authors:** Zhiyan Huang, Xiaolong Li, Qingmiao Zhu, Mengyu Zhu, Yongsheng Fan, Ting Zhao

**Affiliations:** ^1^ The First Affiliated Hospital of Zhejiang Chinese Medical University, Hangzhou, China; ^2^ School of Basic Medical Sciences, Zhejiang Chinese Medical University, Hangzhou, China; ^3^ The Second Affiliated Hospital of Zhejiang Chinese Medical University, Hangzhou, China; ^4^ Key Laboratory of Chinese Medicine Rheumatology of Zhejiang Province, Research Institute of Chinese Medical Clinical Foundation and Immunology, School of Basic Medical Sciences, Zhejiang Chinese Medical University, Hangzhou, China; ^5^ Research Institute of Chinese Medical Clinical Foundation and Immunology, Hangzhou, China

**Keywords:** lupus nephritis, plant extracts, herbal formula, Chinese herbal, immune system, systemic lupus erythematosus

## Abstract

**Ethnopharmacological relevance:**

Lupus nephritis (LN) is an immune complex glomerulonephritis commonly associated with systemic lupus erythematosus. Traditional Chinese medicine (TCM) has emerged as a promising adjuvant therapy for LN, due to its low toxicity and diverse therapeutic effects for long-term management.

**Materials and methods:**

A comprehensive search of PubMed and Web of Science was conducted up to 7 June 2024, using keywords related to lupus nephritis, traditional Chinese medicine, immune cells, and kidney resident cells. Study quality were assessed based on Good Automated Manufacturing Practice guidelines, with evaluations jointly conducted by two authors.

**Results:**

This review includes 31 research papers and summarizes seven herbal formulas and 18 phytochemicals that modulate the autoimmune pathogenesis of LN. Their mechanisms involve regulating immune cells activation, differentiation, apoptosis, as well as influencing resident kidney cells to support renal protection and immune homeostasis. Since TCM exhibit bidirectional regulation, they activate regulatory immune cells while suppress pathogenic factors. Inconsistent or inconclusive findings are discussed.

**Conclusion:**

This review summarizes current research on herbal formulas and phytochemicals in immune cells and kidney resident cells in LN, highlighting the potential and significance of TCM treatment. It also addresses the limitations of existing studies and suggests that future research should focus on exploring the immunosuppressive and kidney-protective effects of herbal formulas and phytochemicals, as well as enhancing the clinical translation and standardization of TCM.

## 1 Introduction

Lupus nephritis (LN) is a type of glomerulonephritis that represents one of the most severe organ manifestations, affecting approximately 50% of the systemic lupus erythematosus (SLE) cases and posing a significant risk for morbidity and mortality (Anders et al., 2020; Parikh et al., 2020). The pathophysiology of LN is heterogeneous, including the deposition of autoantibodies and immune complexes (ICs), the activation and/or proliferation of infiltrating immune cells and kidney resident cells, and the presence of various pathogenic molecules at the site of injury (Davidson, 2016; Lech and Anders, 2013; Mohan et al., 2023).

Conventional treatments encompass steroid therapy, cyclophosphamide (CYC), and mycophenolate mofetil (MMF), while alternative therapies, including anti-BLYS agents, novel calcineurin inhibitors, CD20 blockade, and anti-interferon agents have also been explored (Askanase et al., 2023; Desai et al., 2024). These advancements have enhanced life expectancy and quality, but there has not been significant progress in improving renal failure and mortality rates (Nossent et al., 2024; Parikh et al., 2020). Besides, evidences proved that these drugs can induce cytotoxicity through immunosuppressive and pharmacologic effects, resulting in side effects such as amenorrhea/ovarian failure, cytopenia, and serious infections (Singh et al., 2016a; Singh et al., 2016b). This highlights the urgent need for multi-target therapy and the development of better long-term treatment options or strategies for LN.

The quest for novel therapies, as well as the fact that LN being more common and severe in East Asians than in African-Americans (Petri et al., 2023), have spurred the exploration of TCM. TCMs exhibit diverse and integrated pharmacological effects that help mitigate the progression of LN. Moreover, their ability of reducing adverse side effects makes them a valuable option of long-term adjuvant therapy (Dou et al., 2023). Evidence indicates that combining herbal formulas and phytochemicals such as Shenqi Dihuang decoction, Liuwei Dihuang pill, and Astragalus injection with conventional treatments like cyclophosphamide (CTX) and glucocorticoids (GC) enhances clinical efficacy and reduces SLE disease activity scores more effectively than CTX and GC alone. Moreover, this combined treatment approach has been associated with a lower risk of adverse reactions such as infection, gastrointestinal discomfort, and insomnia (Li et al., 2024), as well as a slightly higher likelihood of reducing or discontinuing glucocorticoid dosage (Li et al., 2014). Additionally, botanical drugs like Cordyceps and artemisinin may help prevent the recurrence of LN and protect kidney function (Lu, 2002).

This review uniquely integrates immune metabolic regulation with the therapeutic potential of TCM in LN, providing a comprehensive analysis of recent advances in preclinical studies. By systematically summarizing the molecular mechanisms and therapeutic implications, this review offers new insights into TCM’s role in LN treatment and identifies key directions for future research. All the herbal formulas and phytochemicals discussed in this review are within the domain of traditional Chinese medicine.

## 2 Methods

In this review, experimental research (*in vitro* and *in vivo* studies) published or available for early access up to 7 June 2024, were retrieved from databases such as PubMed and Web of Science. The search terms included *lupus nephritis*, *traditional Chinese medicine* (e.g., herbal prescriptions, Chinese herbal drugs, plant extracts, herbal extracts), *immune cells* (e.g., T lymphocytes, B lymphocytes), and *kidney-resident cells* (e.g., Podocytes, Mesangial cells).

The inclusion criteria were as follows: (1) studies related to the specified keywords, either individually or in combination, focusing on the therapeutic effects or mechanisms of TCM in LN; (2) studies providing experimental data from *in vitro* or *in vivo* research with detailed methodologies; and (3) full-text articles published in English.

The exclusion criteria were: (1) duplicate search results; (2) studies deemed irrelevant based on title and abstract screening; (3) records lacking mechanistic insights or providing insufficient information; and (4) purely theoretical or review articles.

Based on these criteria, a total of 31 studies investigating the mechanisms of Traditional Chinese Medicine in the treatment of LN were included in this review. The quality and methodological rigor of these studies were assessed using the criteria outlined by the Good Automated Manufacturing Practice (GAMP) best practice guidelines (https://ga-online.org/best-practice/). The evaluation was jointly conducted by authors HZY and LXL. The detailed evaluation results are provided in the supplementary materials.

## 3 Cellular pathogenesis of lupus nephritis

### 3.1 T cells

#### 3.1.1 T cells activation

T cells constitute the majority of kidney-infiltrating immune cells in patients with LN and in lupus-prone mice, and are implicated in the development of progressive kidney failure (Linke et al., 2022; Mohan et al., 2023). T cells can either cause direct cytotoxicity or recruit other inflammatory cells, such as monocytes/macrophages, playing a crucial role in the pathogenesis of experimental and human LN. Studies have revealed that Triptolide, a diterpenoid triepoxide derived from the botanical drug Tripterygium wilfordii Hook. f., inhibits lymphocyte activation and T-cell expression of interleukin-2 at the transcriptional level, positioning it as one of the few immunosuppressants acting at the early stage of T-cell activation signaling (Qiu and Kao, 2003). This characteristic classifies triptolide as operating within a category of immunosuppressants similar to CYC and tacrolimus (Qiu et al., 1999). Furthermore, unlike CYC and tacrolimus, triptolide inhibits both Ca^2+^-dependent and Ca^2+^-independent pathways, thereby affecting T-cell activation through CD28 co-stimulation, indicating that triptolide has broader immunosuppressive effects in the treatment of conditions involving CYC-resistant T-cell activation (Gu et al., 2016; Song et al., 2023). Although there is a lack of studies on other botanical drugs, this example suffices to illustrate the broad potential of TCM in the treatment of LN, particularly as a supplement to targeted therapy.

#### 3.1.2 T cells differentiation

In LN, dysregulated CD4^+^ T cell subsets include Th1, Th17, Tfh, and Treg cells. Th1 and Treg cells are decreased, while Th2, Th17, Tfh17, and Tfh cells are increased. A strong correlation between Th17 and Treg cells with renal involvement was observed (Yuan et al., 2022). These immunological mechanisms are central to TCM-related research and will be discussed further.

##### 3.1.2.1 CD4^+^ Th1 T cells

Numerous clinical and experimental findings have demonstrated a pathogenic role of imbalance towards Th1 cell-mediated immune responses in LN (Chan et al., 2006; Fakhfakh et al., 2022; Linke et al., 2022), which is partly attributed to high glomerular expression of interleukin-12 (IL-12) and interleukin-18 (IL-18) (Liu et al., 2012; Tucci et al., 2010). However, TCM has demonstrated the ability to alter this cellular population imbalance. Hachimi-jio-gan (Ba-Wei-Di-Huang-Wan, HMG) modulated an imbalance toward Th1 predominance in MRL/lpr mice by inhibiting IL-12 production and ameliorating autoimmune disorders (Furuya et al., 2001). Additionally, the Th1 axis was suppressed and the Th2 axis became predominant in Sairei-to-treated MRL/lpr mice, possibly due to an increase in IL-4-producing cells and suppression of IFN-γ expression (Ito et al., 2002). Furthermore, Antroquinonol (Tsai et al., 2012) and DCB-SLE1 (Tsai et al., 2011) have been shown, in mouse models, to reduce renal production of IL-18, thereby facilitating the differential regulation of T cells in treatment. They also exhibit the ability to inhibit local renal inflammation by suppressing NF-κB activation.

##### 3.1.2.2 CD4^+^ Th17 T cells

The level of Th17 cells was found to be higher in LN patients (Fakhfakh et al., 2022). Several reports indicate that interleukin-17 (IL-17) and Th17 cells play important roles in the pathogenesis of LN, with PP2A, ROCK, CREM, and CaMK4 pathways shown to facilitate IL-17 production in SLE (Koga et al., 2017). Celastrol inhibited phospho-STAT3 expression in cultured Th17 cells and upregulated phospho-STAT5 expression in induced regulatory T (iTreg) cells, thereby suppressing Th17 cell induction and promoting iTreg cell generation. It also reduced IL-17 expression in Th17 cells compared with untreated cells (Astry et al., 2015; Zhang et al., 2018). Furthermore, studies have shown that menthone could serve as a potential antirheumatic phytochemical, as its effect on regulating the number of Th1 and Th17 cells and inhibiting the release of pro-inflammatory cytokines, including TNF-α, IL-1β, and IL-6, has been confirmed (Chen X. et al., 2022). DCB-SLE1 has been shown to suppress IL-6 and IL-17 production in an accelerated severe LN model (Tsai et al., 2011), thereby regulating the number of Th17 cells. Administration of lipopolysaccharide resulted in a mixed Th1, Th2, and Th17 response in normal mice, and this effect was inhibited by *in vivo* Tetrandrine (Zou et al., 2019).

Interestingly, Jakiela et al. claimed that Th17 expansion was not related to LN activity, renal histology, or blood and urine inflammatory biomarkers, but was associated with a higher cumulative dose of CYC (Jakiela et al., 2018). This suggests that the use of TCM may help reduce the occurrence of side effects from chronic immunosuppressive therapy and improve treatment efficiency.

##### 3.1.2.3 CD4^+^ Treg cells

Except for the germinal center-Tfr/Tfh imbalance mentioned above, it has been more commonly observed in the pathogenesis of LN that there are fewer Treg cells in LN patients’ peripheral blood than healthy individuals’, with a considerable increase in Th17 cell-to-Treg cell ratios (Li et al., 2022b).

Several have been shown to influence Treg cells in LN. Astragaloside IV (AST IV) has the ability to markedly increase the Foxp3 expression as well as IL-10 and TGF-β secretion levels in Treg in a dose-dependent manner (Li et al., 2016). Antroquinonol can enhance Treg cell suppression in accelerated severe LN (Tsai et al., 2012). Baicalin induces Foxp3 protein expression in cultured T cells, promotes Treg cell differentiation and regulatory activity. It also restores Foxp3 expression following its initial IL-6-mediated inhibition (Yang et al., 2012).

Apart from LN, the abnormal Treg/Th17 ratio is involved in the pathogenesis of many immune-mediated inflammatory diseases. Xu, etc., reviewed that there were nine active ingredients (including Oxymatrine, Baicalin, Triptolide, Paeoniflorin, Sinomenine, Celastrol, Emodin, Diosgenin and Chlorogenic acid) originating from TCM reported to have an immunological regulatory effect on the Th17/Treg axis in IMID treatment (Xu et al., 2020). The mechanism of action of these botanical drugs need to be further explored. The immune-modulating mechanisms on Th17/Treg axis by TCM might provide a broader insight into the treatment of IMID.

##### 3.1.2.4 CD4^+^ Tfh cells

Follicular helper CD4 T (Tfh) cells play a significant role in germinal center formation, B-cell development, affinity maturation, and immunoglobulin class switching (Crotty, 2011; Fazilleau et al., 2009; Zhu et al., 2016). IL-21, a critical cytokine produced by Tfh cells, potently stimulates the differentiation of B cells (King et al., 2008). Tfh cells have been found to increase in LN patients and are associated with B cell activation as well as *in situ* inflammation (Arazi et al., 2019; Liarski et al., 2014; Yuan et al., 2022).

Follicular regulatory T (Tfr) cells, also located in the germinal center and sharing phenotypic characteristics with Tfh cells and Treg cells, inhibit Tfh cells mediated B cell responses (Xu et al., 2017; Zhu et al., 2016). Tfr cells are reported to be reduced in patients with SLE compared with healthy individuals, but this reduction is corrected after standard-of-care treatment (Liu et al., 2018; Xu et al., 2017). Additionally, a progressive reduction in Tfr cells and decreased Tfr/Tfh ratio despite increased Tregs in the renal lymph nodes of NZBWF1/j mice were repored (Kumar et al., 2022).

**FIGURE 1:**
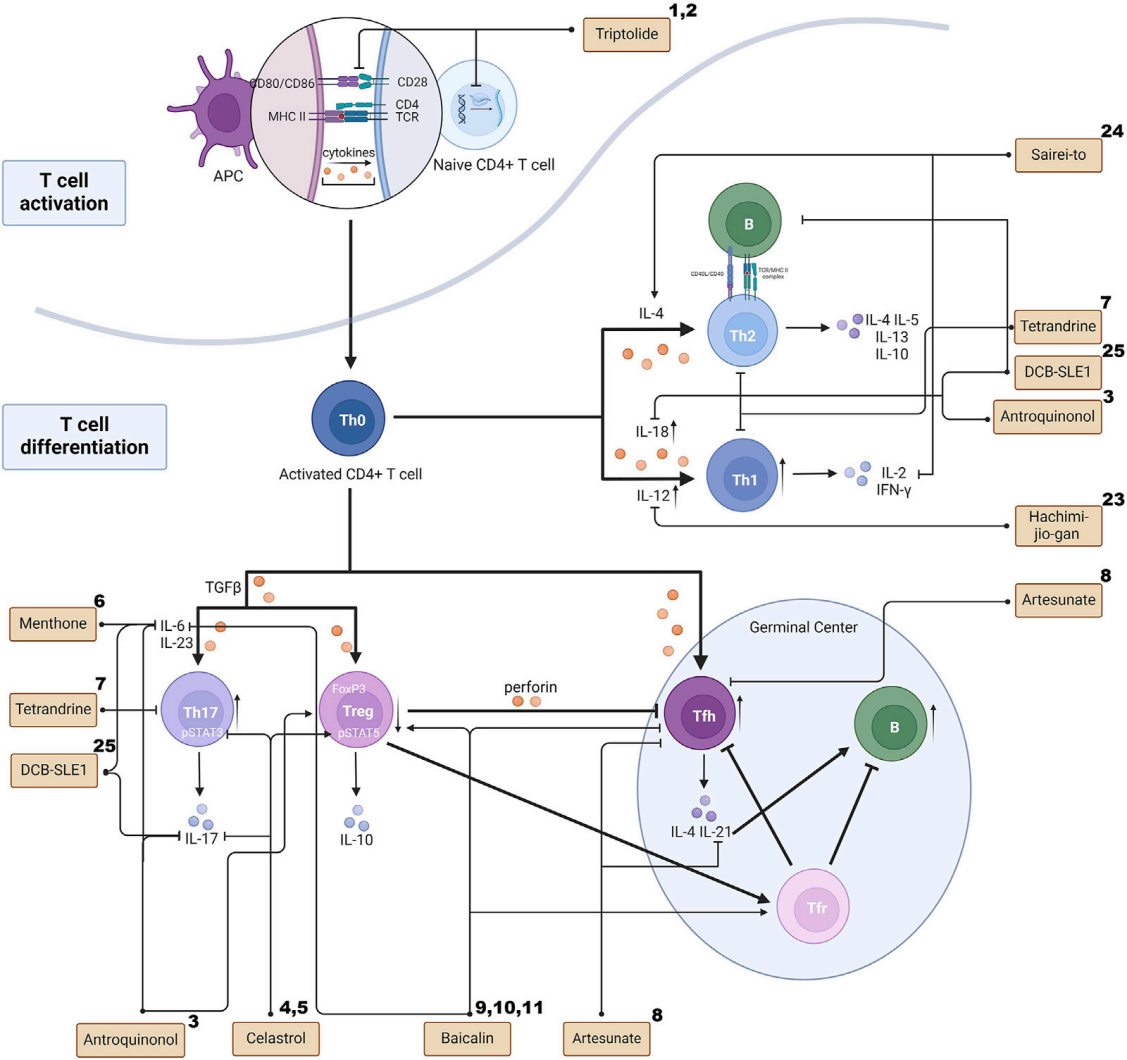
The effects of TCM on T cell activation, differentiation and interaction. The superscript numbers of the herbal formulas/phytochemicals correspond to the reference numbers in Tables 1, 2. Created with BioRender.com.

Artesunate (ART) has shown therapeutic effects by reducing the number of Tfh cells and maintenance of the ratio of Tfr to Tfh in the spleen of MRL/lpr mice (Dang et al., 2019). Baicalin has the ability of inhibiting Tfh cell differentiation and IL-21 production by mTOR activation inhibition, and it can promote Foxp3^+^ regulatory T cell differentiation, including Tfr cells (Yang et al., 2019). Although studies of the balance of Tfr and Tfh cells have been sparse, TCM has shown great therapeutic potential in this regard. (Figure 1 illustrates the differentiation and interaction of the T cell family and the effect of TCM on these processes.).

### 3.2 B cells

#### 3.2.1 B cells and plasma cells

SLE is characterized by the loss of B-cell tolerance and presence of autoantibodies. Anti-dsDNA antibodies cross-react with several renal cell types and are thought to be central to the nephritis process (Tsokos et al., 2016). Researches has focused on the ability of TCM to reduce of autoreactive B cells. Tripterygium wilfordii Hook F (TWHF) regulates the functions of 24 differentially expressed genes (including HMOX1, ALB, and CASP1) through hydrogen bonding, mainly concentrated in the B-cell signaling pathway (Cai et al., 2023). Sairei-to downregulates the proportion of CD19 and the serum levels of IgG1 in MRL/lpr mice, thereby suppressing B cell function (Ito et al., 2002).

Additionally, the upstream regulation of B cells and the role of B cell subsets in the pathogenesis of LN have been further studied. B-cell activating factor (BAFF) and a proliferation-inducing ligand (APRIL) are the most important factors involved in the maturation and activation of B cells (Samy et al., 2017). A study shows that Langchuangping Granule (LG) could attenuate the renal injury via suppressing sBAFF level and BAFF mRNA expressions (Li et al., 2012). Jieduquyuzishen prescription (JP) can downregulate the BAFF/BAFF-R signaling pathway as effectively as GC and suppress proliferation and survival of lymphocytes activated by mBAFF(Wu et al., 2015). BAFF and APRIL are ideal targets for LN treatment, and the value of TCM in this area requires further exploration.

B cell subsets that play a role in the pathogenesis of LN include age-associated B cells (ABCs) and kidney-infiltrated plasma cells (Espeli et al., 2011; Mohan et al., 2023; Rubtsova et al., 2017). Peripheral blood ABCs are increased compared with healthy controls in patients with SLE and strongly correlate with anti-chromatin antibody levels and track with disease activity (Ramsköld et al., 2019; Wang et al., 2018). Infiltrated plasma cells are often present in the renal medulla in LN patients, especially proliferative and membranous types. These cells had the phenotypic characteristics of fully differentiated plasma cells and, similar to long-lived bone marrow plasma cells, they are not in cell cycle (Espeli et al., 2011). However, there is a lack of research on the relationship between TCM and these B cell subsets. More detailed and in-depth researches are needed.

#### 3.2.2 Bregs

Evidences has shown that the percentages of Bregs and their secretion of IL-35 and IL-10 are significantly decreased in LN patients (Heinemann et al., 2016; Watanabe et al., 2010; Xiong et al., 2022). A decrease has been observed in CD72, a regulatory receptor on B cells, while the addition of soluble semaphorin 3A can improve its expression (Vadasz et al., 2014). Although there are no direct studies on the effects of TCM on Breg cells in LN, related research has demonstrated the role of TCM in this context. Depigmented-polymerized phleum pratense (DPG-POL-Phl p) was prominent at inducing IL-10^+^CD19^+^CD5^hi^ and IL-10^+^CD19^+^CD5^hi^CD38^int^CD24^int^ Breg cell subsets (Layhadi et al., 2023). Additionally, administration of Yupinfeng San, a traditional Chinese medical formula, can induce propionic acid production by intestinal bacteria to stabilize IL-10 expression in Breg cells (Zhou et al., 2021) or restore the immune suppressor function of Bregs by inhibiting the expression of Bcl2L12 (Zhou et al., 2019). S. baicalensis extract and its metabolites, baicalin and baicalein, can induce semaphorin 3A expression (Yoshioka et al., 2021), thereby potentially restoring B cells’ regulatory functions by upregulating IL-10 expression (Eiza et al., 2023).

Although IL-10 is known as a potent anti-inflammatory cytokine (Mollazadeh et al., 2019; Saraiva and O’Garra, 2010) and has been emphasized in Breg cell-related therapy, excessive IL-10 levels may lead to immune disorders and increase the risk of autoimmune diseases by disrupting the immune balance and regulating the activation of immune cells. For example, research has showen that IL-10 can increase CD8^+^ T cell infiltration in tissue, induce IFN-γ production, and favor effective T cell memory responses (Saraiva et al., 2020). Recent evidence indicates that IL-10 plays dual roles in SLE: it may inhibit pro-inflammatory effector functions but also seems to be a main driver of the extrafollicular antibody response, promoting direct differentiation of activated B cells into plasma cells (Biswas et al., 2022). Despite the emphasis on promoting Breg cells and IL-10, there are also studies on inhibiting IL-10 using TCM. For example, Wogonin suppresses IL-10 production in B cells via inhibition of the STAT3 and ERK signaling pathways and reduce mRNA and protein levels of the transcription factor Hif-1α(Fan et al., 2020).

**FIGURE 2:**
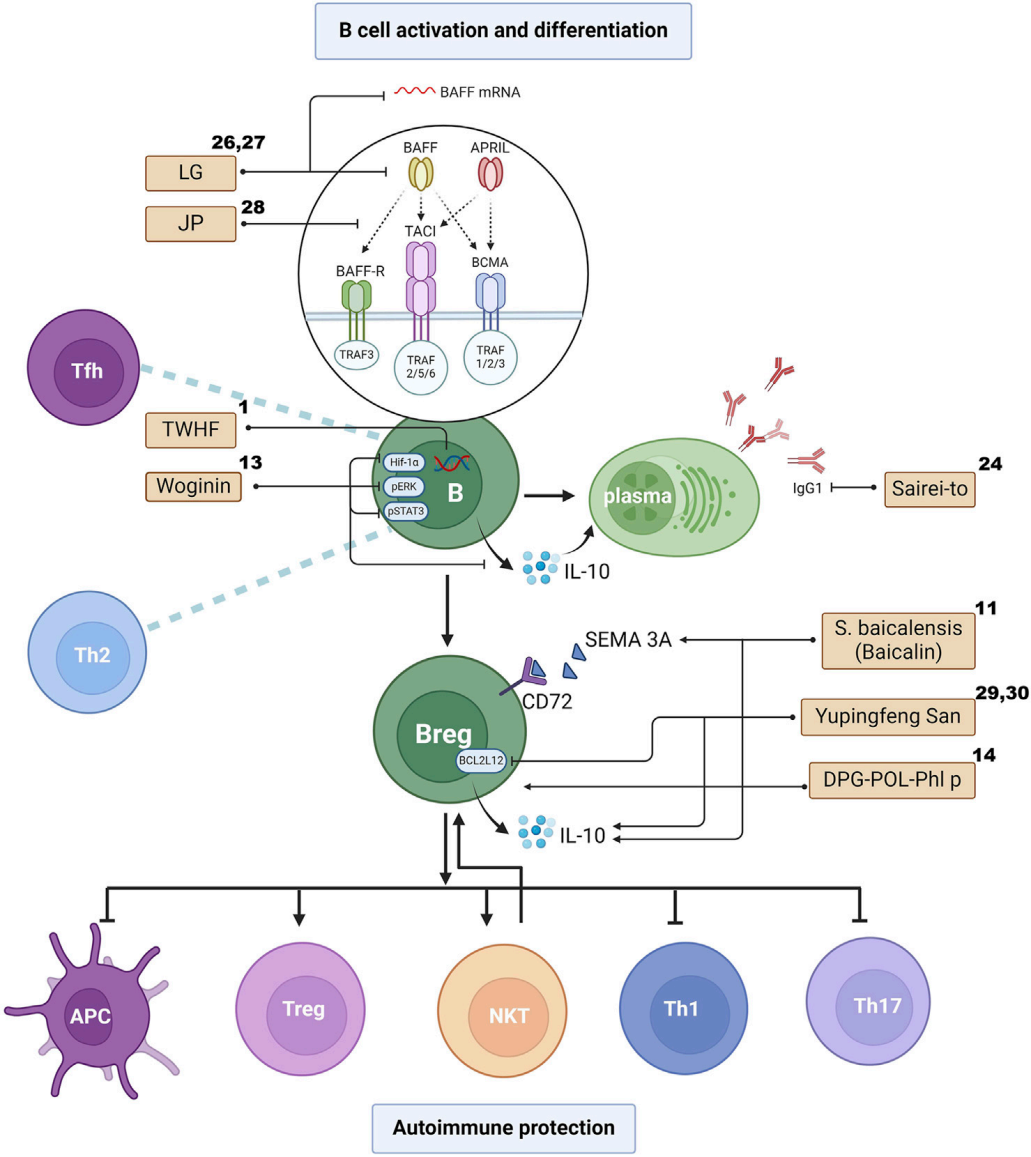
The effects of TCM on B cell activation, differentiation and interaction. The superscript numbers of the herbal formulas/phytochemicals correspond to the reference numbers in Tables 1, 2. Created with BioRender.com.

The aforementioned studies have indicated that TCM may play a role in inducing Bregs and their regulatory factors in LN treatment. On the other hand, due to the pleiotropic effects of cytokines, herbal formulas particularly, offers a more comprehensive regulatory effect owing to its ambiguous targeting, which may reduce side effects and promote positive regulation. Therefore, the therapeutic role of TCM warrants more attention and further exploration. (Figure 2 illustrates the differentiation and interaction of the B cell family and the effect of TCM on these processes.).

### 3.3 NK cells and NKT cells

In SLE patients, circulating levels of Natural Killer (NK) cells were diminished and their cytotoxicities were impaired (Park et al., 2009). Active nephritis in SLE is substantially associated with ILT2^+^ NKT cell and Ki67^+^ NK cell frequencies (Chen et al., 2023; Hudspeth et al., 2019). Additionally, an elevated ratio of CD56^bright^CD16^−^ to CD57^+^CD56^dim^CD16^+^ NK cell distinguishes renal involvement in SLE (Li et al., 2023). The defective functions of NK cells may be due to changes in apoptosis-related protein expressions, such as reduced expression of TRAIL, Bcl-2, and TNFR1(Liphaus et al., 2024). The decline in NK cells may be related to increased consumption due to autoimmunity, as suggested by the fact that the high expression of MCP-1, a chemokine that induces NK cell migration and activation, thereby facilitating kidney disease-related inflammation in LN (Liu et al., 2023).

Although NK cells are one of the most important lymphocytes, their pathogenic role in LN has not received much attention, and the reason for their reduced quantity and weakened pathogenicity in LN have not been clearly studied. Herbal formulas like DCB-SLE1 have been found to suppress NK cell activity (Tsai et al., 2011). However, studies on the effects of TCM on NK cells in the context of treatment are still lacking.

### 3.4 Neutrophils

Neutrophils accumulate in the kidneys of patients with proliferative LN, and their products and ability to induce other immune cells may contribute to pathogenesis of the disease (Nishi and Mayadas, 2019). The herbal formulas and phytochemicals, such as the DCB-SLE1, Antroquinonol, and Citral, have been shown to reduce neutrophil infiltration in the kidney (Tsai et al., 2011; Tsai et al., 2012; Ka et al., 2015). However, some also provide protective effect on neutrophils. For example, JP can reduce the apoptosis and increase the survival of polymorphonuclear neutrophils (Wu et al., 2015).

**FIGURE 3:**
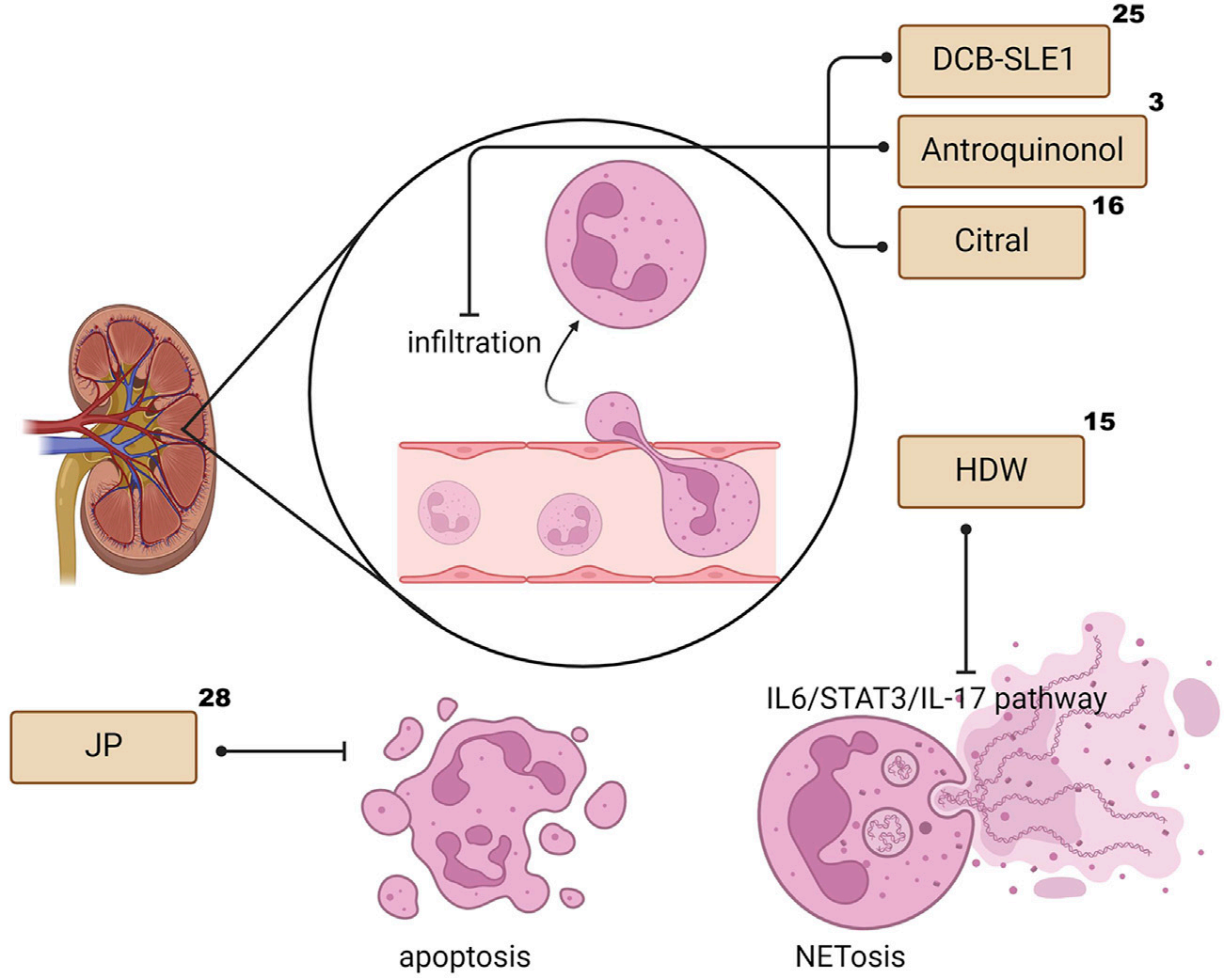
The effects of TCM on Neutrophil infiltration, apoptosis and NETosis. The superscript numbers of the herbal formulas/phytochemicals correspond to the reference numbers in Tables 1, 2. Created with BioRender.com.

Neutrophil extracellular traps (NETs) are fibrous networks that protrude from the membranes of activated neutrophils. Excess NETs damage normal tissues and induce inflammation and immune injury to kidneys (Lee et al., 2017). NETs are elevated in the circulation of patients with SLE, particularly in those with LN, and their abundance correlates with circulating levels of anti-double-stranded DNA (dsDNA), C3 and C4, and proteinuria (Hakkim et al., 2010; van der Linden et al., 2018). Hedyotis diffusa Willd (HDW) treatment has been proven to ameliorate the expression of STAT3, IL-17, Ly6G, and MPO in the kidney and neutrophil Extracellular Trap formation (NETosis), thereby relieving LN (Li et al., 2022a). However, the view that reduced degradation of NETs contributes to LN progression is challenged by experimental data in lupus-prone mice that genetically fail to produce NETs but still suffer from the disease (Kienhöfer et al., 2017; Nishi and Mayadas, 2019). Overall, the role of neutrophils in the pathogenesis of LN and the application of TCM in this field require further study. (Figure 3 illustrates the infiltration, apoptosis and NETosis of the neutrophil family, as well as the effect of TCM on these processes.).

### 3.5 Monocytes and macrophages

Pathogenic macrophages in LN are classified according to source and function. Along with infiltrating monocyte-derived (MoMac) macrophages, the kidneys also have a network of tissue-resident macrophages (TrMac) located around the glomeruli and tubulointerstitium (Mohan et al., 2023). TrMacs orchestrate leukocyte recruitment and are major responders to ICs, while MoMacs take up and present IC antigen (Richoz et al., 2022). Additionally, subpopulations called classically activated macrophages (M1) and alternative activated macrophages (M2) are often discussed in the context of LN mechanism (Chávez-Galán et al., 2015). While it is commonly agreed that kidney macrophages in MRL/lpr mice with spontaneous LN are skewed towards the M1 phenotype rather than the M2 phenotype (Iwata et al., 2012), research has confirmed that M2-phenotype macrophages (CD163^+^) are the dominant subpopulation in human LN. A significant association has been observed among CD163^+^ macrophages, crescents, complement activation, kidney fibrosis and progression to kidney failure (Olmes et al., 2016; Sciascia et al., 2022; Tao et al., 2021).

Macrophage depletion ameliorates nephritis induced by antibodies against the glomerular basement membrane. Demethylzeylasteral (T-96) exhibits reno-protective effects in LN by inhibiting the activation of NF-κB, reducing downstream pro-inflammatory mediators such as TNF-α, COX-2 and ICAM-1, and thus restricting macrophage infiltration (Hu et al., 2015). Similarly, LG downregulates monocyte chemoattractant protein-1 (MCP-1) in BXSB LN mice though the NF-κB signaling pathway (Li et al., 2011). Production of MCP-1 by spleen cells in (NZB x NZW) F1 mice also decreased after triptolide or tripdiolide therapy (Tao et al., 2008). Additionally, Citral alleviates the mouse ASLN model by reducing ATP-induced IL-1β secretion and caspase-1 activation in LPS-primed macrophages (Ka et al., 2015). In the studies related to the treatment of monocyte-macrophages with TCM in LN, many showed that TCM can effectively inhibit the autoimmune response of macrophages, but none have distinguished between macrophage types. As mentioned above, the anti-inflammatory or pro-inflammatory effects and the states in the autoimmune response of different types of macrophages in the development of LN are not consistent, so the therapeutic targets of drugs should be further clarified.

### 3.6 Dendritic cells

In LN, dendritic cells (DC) infiltrate the kidneys, function to present antigens, and organize tertiary lymphoid structures that amplify inflammation (Kurts et al., 2020; Maria and Davidson, 2017), thus playing a critical role in the evolution of LN. when treated with M1, an active metabolite of ginsenoside, the LPS-primed bone marrow-derived dendritic cells (BMDCs) significantly reduced the expression levels of NLRP3, decreased the secretion of pro-IL-1β and p-IκB, and inhibited CD4^+^ T cell proliferation (Lin et al., 2019). Fisetin at non-toxic concentrations suppressed the expression of costimulatory molecules CD80 and CD86, the production of cytokines IL-12, IL-6, and TNF-α, and the endocytic activity of DC during LPS-induced DC maturation (Liu et al., 2010). Bidens pilosa L. petroleum ether extract induced a semi-mature status in DCs, regardless of exposure to a maturation stimulus (Rodríguez Mesa et al., 2023). Additionally, phytochemicals such as Coumarins, Triptolide, Flavonoids from Astragalus membranaceus (Fisch.) Bunge, and Flavonoid luteolin have been confirmed to inhibit the maturation and function of DCs. However, these studies were not performed in specific LN models.

Since DCs are the key link between innate immunity and adaptive immunity and play crucial roles in both promoting immune defense and maintaining immune tolerance (Liu et al., 2021), they are attractive therapeutic targets for autoimmune disease, including LN. For example, evidence reveals that tolerance is observed when partial- or semi-maturation of DCs occurs, and the semi-mature DC phenotype seems to continuously tolerize lymph node T cells against tissue-derived self-antigens or apoptotic cells (Lutz and Schuler, 2002). Hopefully, the therapeutic potential of DCs may bring inspiration for LN treatment with traditional Chinese medicine.

### 3.7 Resident kidney cells

Resident kidney cells, including podocytes, mesangial cells, and tubular epithelial cells have always been considered essential factors in LN. On one hand, damage to these resident kidney cells leads to varying degrees of kidney function loss (Bhargava et al., 2023; Kwok and Tsokos, 2018). On the other hand, inflammatory stimuli to resident cells cause them to produce pro-inflammatory cytokines and present antigen, further aggravating kidney injury (Bhargava et al., 2023; Jamaly et al., 2021; Kwok and Tsokos, 2018; Sakhi et al., 2019).

#### 3.7.1 Podocytes

Reactive oxygen species (ROS) generation can affect the integrity of the podocyte cytoskeleton, resulting in subsequent podocyte detachment from the glomerular basement membrane and onset of proteinuria (Bruno et al., 2023; Guo et al., 2024). Eucarbwenstols A-H could prevent podocyte injury through ROS modulation and regulation of mitochondrial membrane potential (Chen T. et al., 2022), thus potentially having a renoprotective effect.

Aberrant activation of the NLRP3 inflammasome, which might be partly caused by autophagy dysfunction (Biasizzo and Kopitar-Jerala, 2020), plays a significant role in the pathogenesis of LN. Inhibition of NLRP3 has been shown to ameliorate proteinuria, renal histologic lesions, and podocyte foot process effacement (Fu et al., 2017; Ummarino, 2017; Wu et al., 2021). The forementioned M1 can not only reduce ATP mediated ROS production but also inhibit the activation of NLRP3 inflammasome by enhancing autophagy induction in LPS-primed and ATP-activated podocyte (Lin et al., 2019).

Recent findings suggest that podocytes share many elements of the innate and adaptive immune systems (Bhargava and Tsokos, 2019). They produce and express complement components and receptors, as well as major histocompatibility complex and co-stimulatory molecules, which may be involved in local immune events (Moll et al., 2006; Appay et al., 1990). However, the study of TCM in these related fields has not yet been explored.

#### 3.7.2 Mesangial cells

As the mesangium is one of the primary sites for IC deposition, mesangial cells (MCs) constantly undergo severe damage, resulting in excessive proliferation and increased extracellular matrix production (Liu M. et al., 2022). ICAM-1, a cell surface glycoprotein and an adhesion receptor that regulate leukocyte recruitment from circulation to sites of inflammation (Bui et al., 2020), was found in the mesangial area and deposited along the glomerular capillary walls in MRL/lpr mice. The distribution intensity of ICAM-1, immunoglobulins and C3 significantly decreased after treatment with stragalin in form of decoction (Chen et al., 1995). Langchuangjing Granule (LCJ) can also inhibit the increase of serum ICAM-1 content and partially improve plasma distribution, as well as suppress the atrophy of renal corpuscles and the proliferation of mesangial cells (Zhu et al., 2004).

The role of resident kidney cells in the development of LN is becoming more defined and distinct. However, there is a huge gap in the field of TCM treatment for resident kidney cells. Research on cells that play an equally important role in the pathogenesis of LN, such as tubular epithelial cells, has not been conducted. Additionally, the pathogenic capacities of resident kidney cells, such as podocytes’ abilities to present antigens and participate in the formation of crescents, have not been studied in the area of TCM. More recent studies have pointed to the restoration of kidney resident cell function using cell-targeted approaches to prevent and treat LN, and TCM should play a role in this effort. (Tables 1, 2, the plant name has been checked with http://mpns.kew.org/mpns-portal/).

**TABLE 1:** The effects of phytochemicals on LN treatment.

Phytochemical	Origin	Immunocyte/kidney resident cell	Cytokine	Signaling pathway	References
Triptolide	Tripterygium wilfordii Hook.f. [Celastraceae; Tripterygii wilfordii radix]	Inhibit T cells activation	Inhibit T-cell expression of interleukin-2 at the level of transcription	Inhibit both Ca (2+)-dependent and Ca (2+)-independent pathways, therefore affecting T-cell activation through CD28 co-stimulation	1 (Qiu et al., 1999)
		Downregulate monocytes	Downregulate monocyte chemoattractant protein-1 (MCP-1)	Not mentioned	2 (Tao et al., 2008)
Antroquinonol	Antrodia camphorata [Polyporaceae; Antrodiae camphoratae fructus]	Inhibit local renal inflammation	Inhibit IL-18 production	Suppress NF-κB activation	3 (Tsai et al., 2012)
		Suppress T cell proliferation/activation			
		Modulate Th1/Th2 cells cytokines balance			
		Upregulate Treg cells	Suppress IL-6 expression and IL-17 production	Activate Nrf2 pathway	
		Suppress Th17 cells induction			
		Ameliorated development of severe renal lesions, especially cellular crescent formation, neutrophil infiltration, fibrinoid necrosis	Not mentioned		
Celastrol	Celastrus aculeatus Merr. [Celastraceae; Celastri aculeati herba]	Suppress Th17 cells induction	Lower IL-17 expression in Th17	Inhibit phospho-STAT3 expression in Th17 cells	4 (Astry et al., 2015)
		Promote Treg cells generation	Not mentioned	Upregulate phospho-STAT5 expression in Treg cells	5 (Zhang et al., 2018)
Menthone	Mentha × piperita L. [Lamiaceae; Menthae piperitae folium et aetheroleum]	Regulate the number of Th1 and Th17 cells	Inhibit the release of pro-inflammatory cytokines including TNF-α, IL-1β, and IL-6	Not mentioned	6 (Chen X. et al., 2022)
Tetrandrine (TET)	Stephania tetrandra S. Moore [Menispermaceae; Stephaniae tetrandrae radix]	Inhibit the differentiation of proinflammatory Th1, Th2 and Th17 cells	Not mentioned	Inhibit master transcription factors, namely, T-bet, Gata3 and RORγt	7 (Zou et al., 2019)
		Spare the generation of Tregs			
Artesunate (ART)	Artemisia annua L. [Asteraceae; Artemisiae annuae herba]	Reduce the number of Tfh cells	Decrease the levels of pathogenic cytokines (IL-6, IFN-γ and IL-21)	Activate JAK2-STAT3 signaling pathway	8 (Dang et al., 2019)
		Maintain the ratio of Tfr to Tfh cells			
Baicalin	Scutellaria baicalensis Georgi [Lamiaceae; Scutellariae baicalensis radix]	Inhibit Tfh cells differentiation	Inhibit IL-21 production in Tfh	inhibit mTOR activation	9 (Yang et al., 2019)
		Promote Treg cells differentiation and function including part of Tfr cells	Induce Foxp3 protein expression in T cells	Not mentioned	10 (Yang et al., 2012)
		Reconstruct B cells’ regulatory functions by upregulating IL-10 expression	Induce semaphorin 3 A expression	Upregulate CD72-semaphorin3A axis	11 (Yoshioka, 2021)
Astragaloside IV (AST IV)	Astragalus mongholicus Bunge [Fabaceae; Astragali radix]	Promote Treg cells function	Increase the Foxp3 expression as well as IL-10 and TGF-β secretion levels in Treg	Not mentioned	12 (Li et al., 2016)
Wogonin	Scutellaria baicalensis Georgi [Lamiaceae; Scutellariae baicalensis radix]	Suppress B cells function	Suppresses IL-10 production	Inhibit the STAT3 and ERK signaling pathway inhibit mRNA and protein levels of Hif-1α	13 (Fan et al., 2020)
Depigmented-polymerized (DPG-POL-Phl p)	Phleum pratense L. [Poaceae; Phlei pratensis herba]	Induce IL-10 (+)CD19 (+)CD5 (hi) and IL-10 (+)CD19 (+)CD5 (hi)CD38 (int)CD24 (int) regulatory B cells	Increase in IL-10 expression	Not mentioned	14 (Layhadi et al., 2023)
Active metabolites in Hedyotis diffusa Willd (HDW): asiatic acid, neoandrographolide, glycyrrhetinic acid, oleanolic acid, ursolic acid, and wilforlide A		Ameliorate neutrophil NETosis	Ameliorate the expression of STAT3, IL-17, Ly6G, and MPO in the kidney	Inhibit the IL-6 and STAT3/IL-17 signaling pathways	15 (Li et al., 2022a)
Citral	Litsea cubeba (Lour.) Pers. [Lauraceae; Litseae cubebae fructus]	Ameliorate intrinsic cell proliferation, cellular crescents, neutrophil influx, fibrinoid necrosis in the glomerulus, and peri-glomerular infiltration of mononuclear leukocytes			16 (Ka et al., 2015)
		Inhibit macrophages	Reducing ATP-induced IL-1β secretion and caspase-1 activation	Not mentioned	
Demethylzeylasteral (T-96)	Tripterygium wilfordii Hook.f. [Celastraceae; Tripterygii wilfordii radix]	Restrict macrophage infiltration	Reduce the downstream pro-inflammatory mediators such as TNF-α, COX-2 and ICAM-1	Inhibit the activation of NF-κB	17 (Hu et al., 2015)
M1	Panax ginseng C.A.Mey. [Araliaceae; Ginseng radix]	Modulate Th cell activation	Decrease the secretion of pro IL-1β and p-IκB in BMDC	Not mentioned	18 (Lin et al., 2019)
		Induce Treg cell differentiation	Inhibit NLRP3 inflammasome associated with autophagy induction		
		Inhibit CD4^+^ T cell proliferation			
		Protect podocytes	Reduce the ATP-mediated production of ROS	Not mentioned	
			Inhibit the activation of NLRP3 inflammasome by enhancing the induction of autophagy		
Fisetin	Producted widely from botanical drugs	Suppress the endocytic activity of dendritic cells	Suppress the expression of CD80 and CD86	Not mentioned	19 (Liu et al., 2010)
			Suppress the production of IL-12, IL-6, and TNF-α		
Petroleum ether extract of Bidens pilosa L. [Asteraceae; Bidens pilosa herba]		Induce a semi-mature status in DCs	Immature or semi-mature DCs expressing IL-10 facilitated the immune tolerance through inducing Tregs	Not mentioned	20 (Rodríguez Mesa et al., 2023)
		Induce M2 polarization or a hybrid M1/M2 phenotype in MØs			
Eucarbwenstols A-H	Eucalyptus robusta Sm. [Myrtaceae; Eucalypti robustae folium]	Prevents the podocyte cells’ injury	Modulate ROS and regulate mitochondrial membrane potentia	Not mentioned	21 (Chen T. et al., 2022)
Stragalin	Astragalus mongholicus Bunge [Fabaceae; Astragali mongholici radix]	Protect mesangial cells	Decrease the distribution intensity of ICAM-1, immunoglobulins and C3	Not mentioned	22 (Chen et al., 1995)
		Inhibit leukocyte recruitment			

**TABLE 2:** The effects of herbal formulas on LN treatment.

Herbal formula	Ingredient	Immunocyte/kidney resident cell	Cytokine	Signaling pathway	References
Hachimi-jio-gan (Ba-Wei-Di-Huang-Wan, HMG)	Rehmannia glutinosa (Gaertn.) Libosch. ex DC. [Orobanchaceae; Rehmanniae glutinosae rhizoma], Cornus officinalis Siebold & Zucc. [Cornaceae; Cornus officinalis sarcocarpium], Dioscorea oppositifolia L. [Dioscoreaceae; Dioscoreae oppositifoliae rhizoma], Alisma plantago-aquatica subsp. Orientale (Sam.) Sam. [Alismataceae; Alismatis plantago-aquaticae tuber], Poria cocos (Schw.) Wolf [Polyporaceae; Poria], Paeonia × suffruticosa Andrews [Paeoniaceae; Paeoniae suffruticosae cortex], Cinnamomum verum J.Presl [Lauraceae; Cinnamomi verum cortex] and Aconitum carmichaelii Debeaux [Ranunculaceae; Aconiti carmichaelii radix]	Modulate Th1/Th2 cytokines balance	Inhibit IL-12 production	Not mentioned	23 (Furuya et al., 2001)
Sairei-to	Bupleurum chinense DC. [Apiaceae; Bupleuri chinensis radix], Glycyrrhiza uralensis Fisch. [Fabaceae; Glycyrrhizae radix], Cinnamomum cassia (L.) J.Presl [Lauraceae; Cinnamomi cortex], Scutellaria baicalensis Georgi [Lamiaceae; Scutellariae radix], Alisma plantago-aquatica subsp. orientale (Sam.) Sam. [Alismataceae; Alismatis rhizoma], Pinellia ternata (Thunb.) Breit. [Araceae; Pinelliae rhizoma], Polyporus umbellatus (Pers.) Fries [Polyporaceae; Polypori sclerotium], Poria cocos (Schw.) F.A.Wolf [Polyporaceae; Poriae sclerotium], Atractylodes lancea (Thunb.) DC. [Asteraceae; Atractylodis rhizoma], Ziziphus jujuba Mill. [Rhamnaceae; Zizyphi fructus], Panax ginseng C.A.Mey. [Araliaceae; Ginseng radix] and Zingiber officinale Roscoe [Zingiberaceae; Zingiberis rhizoma]	Modulate Th1/Th2 cytokines balance	Enhance IL-4 production	Not mentioned	24 (Ito et al., 2002)
			Suppress the IFN-γexpression		
		Suppress B cells function	Downregulates CD19 and the serum levels of IgG1		
DCB-SLE1	Atractylodes macrocephala Koidz. [Asteraceae; Atractylodis macrocephalae rhizoma], Eucommia ulmoides Oliv. [Eucommiaceae; Eucommiae cortex], *Lonicera japonica* Thunb. [Caprifoliaceae; Lonicerae caulis], and Hedyotis diffusa Willd. [Rubiaceae; Hedyotidis diffusae herba]	Modulate Th1/Th2 cytokines balance	Inhibit IL-18 production	Inhibit Renal NF-B activation	25 (Tsai et al., 2011)
		regulate the number of Th17 cells	suppress IL-6 and IL-17 production		
		Suppress B cell activation	Not mentioned		
		Decrease autoantibody production			
		Inhibits Renal Infiltration of T Cells, Monocytes/Macrophages, and Neutrophils	Not mentioned		
		suppress NK cell activity	Not mentioned		
Langchuangping Granule (LG)	Sophora tonkinensis Gagnep. [Fabaceae; Sophorae tonkinensis radix], Panax notoginseng (Burkill) F.H.Chen [Araliaceae; Notoginseng radix], Ligustrum lucidum Ait. [Oleaceae; Ligustri lucidi fructus], Gentiana macrophylla Pall. [Gentianaceae; Gentianae macrophyllae radix], Arnebia euchroma (Royle) Johnst. [Boraginaceae; Arnebiae radix], herba Radix Saposhnikovia divaricata (Turcz.) Schischk. [Apiaceae; Saposhnikoviae radix], etc.	Suppress B cells function	Suppress sBAFF level and BAFF mRNA expressions	Not mentioned	26 (Li et al., 2012)
		Downregulate monocytes	Downregulate monocyte chemoattractant protein-1 (MCP-1)	Inhibit NF-κB signaling pathway	27 (Li et al., 2011)
Jieduquyuzishen prescription	Artemisia annua L. [Asteraceae; Artemisiae annuae herba], Cimicifuga heracleifolia Kom. [Ranunculaceae; Cimicifugae heracleifoliae rhizoma], Hedyotis diffusa Willd. [Rubiaceae; Hedyotis diffusae herba], Paeonia veitchii Lynch [Paeoniaceae; Paeoniae veitchii radix], Trionyx sinensis Wiegmann [Trionychidae; Trionycis sinensis carapax], *Centella asiatica* (Linn.) Urban [Apiaceae; Centellae asiaticae herba], Citrus medica Linn. var. sarcodactylis (Noot.) Swingle [Rutaceae; Citri medicae var. sarcodactylis fructus], Glycyrrhiza uralensis Fisch. [Fabaceae; Glycyrrhizae uralensis rhizoma], Coix lacryma-jobi L. var. Mayuen (Roman.) Stapf [Poaceae; Coicis semen], Rehmannia glutinosa (Gaert.) Libosch. [Orobanchaceae; Rehmanniae radix preparata]	Suppress proliferation and survival of lymphocytes (B cells included) activated by mBAFF	Not mentioned	Downregulate the BAFF/BAFF-R signaling pathway	28 (Wu et al., 2015)
		Reduce the apoptosis and raise the survival of polymorphonuclear neutrophils			
Yupinfeng San	Astragalus membranaceus (Fisch.) Bunge [Fabaceae; Astragli radix], Atractylodes macrocephala Koidz. [Asteraceae; Atractylodis macrocephalae rhizoma] and Saposhnikovia divaricata (Turcz. ex Ledeb.) Schischk. [Apiaceae; Saposhnikoviae radix]	Restore the immune suppressor function of Breg cells	Stabilize the IL-10 expression in B cells	Induce propionic acid production by intestinal bacteria, which counteracts the effects of Tristetraprolin on inducing IL-10 mRNA decay in B cells through the AKT/T-bet/granzyme B pathway	29 (Zhou et al., 2021)
			Inhibit the expression of Bcl2L12		30 (Zhou et al., 2019)
Langchuangjing Granule (LCJ)	Rehmannia glutinosa (Gaertn.) Libosch. ex DC. [Orobanchaceae; Rehmanniae radix], Leonurus japonicus Houtt. [Lamiaceae; Leonuri herba], Hedyotis diffusa Willd. [Rubiaceae; Hedyotis diffusae herba], Cornus officinalis Siebold & Zucc. [Cornaceae; Corni fructus], Paeonia × suffruticosa Andrews [Paeoniaceae; Moutan cortex], etc.	Suppress the proliferation of mesangial cell	Inhibit the increase of serum ICAM-1 content	Not mentioned	31 (Zhu et al., 2004)

## 4 Discussion

The pathogenesis of LN involves abnormal autoimmune responses, including autoantibody and immune complex deposition, activation of infiltrating immune cells, and damage to kidney resident cells. Traditional Chinese Medicine (TCM) treatments, primarily in the form of herbal formulas and phytochemicals, exhibit distinct therapeutic effects in LN by suppressing autoimmune activity, promoting regulatory functions, and protecting kidney cells. This review explores the role of TCM in modulating immunity and mitigating LN pathogenesis, highlighting its potential value in LN treatment.

Previous reviews on TCM treatment for LN have primarily categorized therapeutic effects based on specific drugs, detailing the mechanisms of herbal formulas and phytochemicals (Dou et al., 2023; Liu L. et al., 2022; Wang et al., 2022). In contrast, this review not only acknowledges previous research findings but also adopts an innovative approach by focusing on LN immunopathogenesis, systematically delineating TCM applications within immune mechanisms. This perspective enhances clarity, provides deeper insights into specific pathways, and offers greater research value for future studies.

However, this review has several limitations:

1.Limited coverage of certain immune cells. While this section provides a comprehensive review of T cells, B cells, neutrophils, monocytes, and dendritic cells, the discussion of others (e.g., NK cells, macrophage subtypes) remains limited due to the lack of relevant research.
2.Lack of mechanistic detail in some areas. Although various immune pathways are mentioned, some mechanisms are only briefly introduced. For example, while the relationship between resident kidney cells and immune responses is acknowledged, the specific ways in which TCM influences kidney cell-mediated immune modulation remain unexplored.
3.Absence of standardized evaluation of TCM effects. This review highlights the potential immunomodulatory effects of several herbal formulas and phytochemicals; however, there is no standardized comparison of their efficacy. Additionally, discussions on dosage, bioavailability, and pharmacokinetics of TCM compounds in LN treatment remain limited, despite their crucial role in clinical translation.
4.Insufficient discussion of clinical relevance. The review predominantly focuses on preclinical (*in vitro* and *in vivo*) studies, while clinical evidence for TCM interventions is scarcely addressed due to the diversity of herbal formulas and phytochemicals.
Therefore, the following directions for future research are proposed:
1.Expanding the study of immune cell subtypes and kidney resident cells. The autoimmune mechanisms underlying LN remain unclear, and research on TCM in LN is still incomplete and often idealized. Further investigation is needed into the roles of NK cells, specific macrophage subtypes, dendritic cells, and various kidney resident cells in LN pathogenesis, as well as their modulation by TCM.
2.Mechanistic exploration of TCM effects. Future studies should elucidate the precise molecular pathways through which TCM regulates immune responses in LN, particularly in relation to cytokine signaling, metabolic regulation, and epigenetic modifications.
3.Integration of TCM with conventional LN therapies. Research should explore the synergistic effects of TCM with standard immunosuppressive treatments (e.g., corticosteroids, MMF, anti-BAFF therapy) to assess whether TCM can enhance efficacy or mitigate side effects.
4.Clinical translation and standardization. More clinical trials are required to validate the efficacy and safety of TCM formulations. Establishing standardized dosages and assessing the bioavailability of TCM monomers will facilitate their clinical application.

## 5 Conclusion

This review offers insights into the cellular mechanisms of LN while primarily clarifying the immunological basis for selecting herbal formulas and phytochemicals of TCM origin in LN therapy. However, future research should address gaps in immune cell coverage, mechanistic insights, clinical applicability, and pharmacological standardization to improve the translational potential of TCM in LN treatment. With continued in-depth investigation, the development of more efficient, effective, and low-toxicity herbal formulas and phytochemicals for LN treatment is achievable.

## Glossary

LN: lupus nephritis
SLE: systemic lupus erythematosus
TCM: traditional Chinese medicine
CYC: cyclophosphamide
MMF: mycophenolate mofetil
GC: glucocorticoids
Th1 cell: T helper 1
Th17 cell: T helper 17
Tfh cell: T follicular helper
Treg cell: T regulatory
iTreg cell: induced regulatory T
ABCs: age-associated B cells
MoMac: monocyte-derived macrophages
TrMac: tissue-resident macrophages
M1: classically activated macrophages
M2: alternative activated macrophages
NK cell: Natural Killer
DC: dendritic cells
BMDC: bone marrow-derived dendritic cell
MC: mesangial cell
IL-17: interleukin-17
IL-21: interleukin-21
IL-4: interleukin-4
IL-12: interleukin-12
IL-18: interleukin-18
IL-1: interleukin-1
IL-6: interleukin-6
IL-10: interleukin-10
IL-35: interleukin-35
IL-17: interleukin-17
NETs: Neutrophil extracellular traps
NETosis: Neutrophil Extracellular Trap formation
AST IV: Astragaloside IV
BAFF: B-cell activating factor
APRIL: a proliferation-inducing ligand
ROS: Reactive oxygen species
MCP-1: monocyte chemoattractant protein-1
TWHF: Tripterygium wilfordii Hook F
LG: Langchuangping Granule
JP: Jieduquyuzishen prescription
DPG-POL-Phl p: Depigmented-polymerized phleum pratense
T-96: Demethylzeylasteral
LCJ: Langchuangjing Granule.
